# Indoor Air Pollution, Nighttime Heart Rate Variability and Coffee Consumption among Convenient Store Workers

**DOI:** 10.1371/journal.pone.0063320

**Published:** 2013-08-15

**Authors:** Kai-Jen Chuang, Hsiao-Chi Chuang, Lian-Yu Lin

**Affiliations:** 1 Department of Public Health, School of Medicine, College of Medicine, Taipei Medical University, Taipei, Taiwan; 2 School of Public Health, College of Public Health and Nutrition, Taipei Medical University, Taipei, Taiwan; 3 Division of Pulmonary Medicine, Department of Internal Medicine, Shuang Ho Hospital, Taipei Medical University, Taipei, Taiwan; 4 School of Respiratory Therapy, College of Medicine, Taipei Medical University, Taipei, Taiwan; 5 Department of Internal Medicine, Division of Cardiology, National Taiwan University Hospital, Taipei, Taiwan; University of Sao Paulo, Brazil

## Abstract

**Background:**

The association between ambient air pollution and heart rate variability (HRV) has been well-documented. Little is known about the association of HRV at night with indoor air pollution and coffee consumption. The aim of this study was to investigate the association of HRV indices with indoor air pollution, working time and coffee consumption.

**Methods:**

We recruited 60 young healthy convenient store workers to monitor indoor PM_2.5_ (particulate matter with an aerodynamic diameter ≤ 2.5 µm) exposures, coffee consumption (yes vs. no) and HRV indices during daytime (0700–1500 hours) and nighttime (2300-0700 hours). We used linear mixed effects models to assess the associations of HRV indices with indoor PM_2.5_ exposures and coffee consumption.

**Results:**

We observed the inverse association between indoor PM_2.5_ exposures and HRV indices, with a decrease in all HRV indices with increased indoor PM_2.5_ exposures. However, the decrease was most pronounced during nighttime, where a 1 interquartile range (IQR) increase in indoor PM_2.5_ at 4-hr time-weighted moving average was associated with a change of −4.78% 5-min standard deviation (SD) of normal-to-normal intervals for 5-min segment (SDNN) and −3.23% 5-min square root of the mean squared differences of successive intervals for 5-min segment (r-MSSD). Effects of indoor PM_2.5_ were lowest for participants with coffee consumption during daytime.

**Conclusions:**

Indoor PM_2.5_ exposures were associated with decreased 5-min SDNN and 5-min r-MSSD, especially during nighttime. The effect of indoor PM_2.5_ on HRV indices may be modified by coffee consumption in young healthy convenient store workers.

## Introduction

The association between indoor ambient air pollution and cardiovascular effects has been reported in previous studies [1], [2]. The plausible pathophysiologic mechanisms of pollution-induced cardiovascular effects involve cardiac autonomic dysfunction [3] represented by using heart rate variability (HRV). Decrease in HRV has been linked to increased risk of hypertension [4], the rapid progression of atherosclerosis [5] and cardiovascular mortality [6]. Several studies have described the association of decreased HRV with ambient air pollution [7]–[9] by using various methods because of uncertainty in time course of HRV responses to ambient air pollution exposure. The majority of studies have examined changes in short-term HRV with ambient air pollution exposure averaged over hours or days [10]. However, no study has investigated the association between occupational exposure to indoor air pollutants and HRV among young healthy workers in nighttime.

Caffeine is the most widely consumed substance in coffee, tea and cocoa around the world [11]. The main effects of caffeine include increased respiratory rate, diuresis and psychoactive effects on autonomic function [12]. It has been shown that caffeine may be associated with arrhythmia [13] and hypertension [14]. A recent study further reported the association between coffee consumption and HRV alteration in young, healthy people [15]. Although the above mentioned studies indicated the effects on HRV exerted by caffeine in a beverage, little is known about the interaction between coffee consumption, indoor air pollutants exposures and HRV alteration. The aim of this study was to investigate the association of HRV with occupational exposure to indoor air pollutants and coffee consumption among young healthy convenient store workers. We further investigated whether coffee consumption modified effects of indoor air pollutants and whether workers were vulnerable to indoor air pollutants effects in nighttime.

## Materials and Methods

### Ethics Approval

This study was approved by the ethics committee of St. Mary’s Medicine Nursing and Management College. An written informed consent was obtained from each participant before the study.

### Participants and Study Design

The study participants consisted of 60 young, healthy, non-smoking students aged 19 to 30 working in various convenient stores in the Taipei metropolitan area. The selection criteria of study participants were as follows: students aged between 18 and 30; no history of smoking; no medication that might affect cardiac rhythm; and no cardiovascular diseases, such as coronary artery disease, arrhythmia, hypertension, diabetes mellitus, and dyslipidemia. The statuses of work shift and coffee consumption were also used to divide recruited participants into different groups. Half of the participants worked in daytime (0700–1500 hours), whereas the other 30 participants worked in nighttime (2300-0700 hours). The protocol included three visits that entailed continuous 8-hour resting electrocardiographic (ECG), indoor air pollution, noise and meteorological conditions monitoring at approximately 1-week intervals in the years 2009 to 2012. Each of the 60 participants had 3 visits, making a total of 180 visits. During their first visits, age, sex, body mass index (BMI), environmental characteristics were recorded by a questionnaire. Information on coffee consumption, diet and time-activity patterns during the study were collected in all visits.

### Indoor Air Pollution, Noise and Meteorological Data

We conducted 8-hour continuous indoor air pollution, noise and meteorological conditions monitoring each visit for each participant. Indoor PM less than 2.5 µm in diameter (PM_2.5_), temperature and relative humidity were measured continuously using a personal dust monitor (DUST-check portable dust monitor, model 1.108; Grimm Labortechnik Ltd., Ainring, Germany), which measured and recorded 1-minute mass concentrations of PM_2.5_ as well as temperature and relative humidity. Total volatile organic compounds (VOCs) were measured continuously using a total VOC monitor (ppbRAE Plus, model PGM-7240; RAE Systems, Inc., San Jose, CA, USA). Carbon dioxide (CO_2_) and carbon monoxide (CO) concentrations were determined with an electrochemical sensor (Q-Trak monitor, model 8554, TSI, Inc., Shoreview, MN, USA). Noise level was measured by using a portable noise dosimeter (Logging Noise Dose Meter Type 4443, Brüel &Kjær, Nærum,Denmark.), which reports 1-min continuous equivalent sound levels(Leq) and the time-weighted-averages (TWA) of noise doses. The range of 30–100 dBA was used to measure noise exposure with 1-min readings over 8 hours. After sampling, the raw data for 1-min indoor PM_2.5_, total VOCs, CO_2_, CO, temperature, relative humidity and noise measurements were matched with the sampling time of HRV monitoring and then computed to 1- to 4-hr time-weighted moving averages if 75% of the data were present.

### HRV Monitoring

We performed continuous ambulatory electrocardiographic (ECG) monitoring using a PacerCorder 3-channel device (model 461A; Del Mar Medical Systems LLC, Irvine, CA, USA) with a sampling rate of 250 Hz (4 msec). ECG tapes were analyzed using a Delmar Avionics model Strata Scan 563 (Irvine, CA). A complete 5-minute segment of NN interval was taken for HRV analysis, including standard deviation of normal-to-normal (NN) intervals (SDNN) and the square root of the mean of the sum of the squares of differences between adjacent NN intervals (r-MSSD).

### Statistical Analysis

The mixed-effects regression models were applied to examine the association of indoor air pollutants with log_10_−transformed HRV indices by running R statistical software version 2.15.1. The independent variables were indoor PM_2.5_, total VOCs, CO_2_ and CO at 1- to 4-hr time-weighted moving averages, whereas the dependent variables were SDNN and r-MSSD. We treated participant’s sex, age, BMI, order of the visit, hour of day, temperature, relative humidity, noise and indoor air pollutants as fixed effects and fitted participant identity number as a random intercept term in our mixed-effects models. Effect modification by coffee consumption (yes versus no) and working time (daytime versus nighttime) were assessed in separate linear mixed effects models by including interaction terms between indoor air pollution effects and each potential effect modifier. Pollution effects are expressed as percent changes by interquartile range (IQR) changes as [10(β× IQR) −1] × 100% for log_10_−transformed HRV indices, where β is the estimated regression coefficient.

## Results

Sixty young convenient store workers s were recruited for this study, and 180 workplace visits with 8640 hourly environmental and physiological measurements were included in data analyses. As shown in Table 1, the ages of 60 study participants were 19–30 years among 30 daytime participants and 21–30 years among 30 nighttime participants. Their mean BMIs were 21.5 kg/m^2^ and 22.5 kg/m^2^, respectively. Half of them consumed coffee during the study. The amounts of coffee consumed by these participants were 12–16 oz/visit. No participant consumed tea, cocoa or chocolate. The male/female ratios were 1∶1 in both groups. The nighttime group had significantly higher values of HRV indices than the daytime group. All participants’ indoor air pollutants exposures, noise and meteorological conditions during the study period were summarized in Table 2. The daytime participants had significantly higher indoor PM_2.5_, Total VOCs and noise exposures than the nighttime participants. No significant difference between daytime and nighttime study periods for CO and CO_2_ exposures. The workplace weather conditions were pleasant with a temperature range of 24.6 to 25.2°C and a relative humidity range of 55.7 to 56.4% during the study period.

**Table 1:** Basic characteristics of 60 study participants (mean ± standard deviation).

Characteristics	Daytimeparticipants	Nighttimeparticipants	P value^a^
Sex, n			–
Female	15	15	
Male	15	15	
Age, y			–
Mean	22.1±1.1	23.4±1.4	
Range	19–30	21–30	
Body mass index, kg/m^2^			–
Mean	21.5±1.8	22.5±2.2	
Range	18.5–26.4	20.6–28.2	
Log_10_ SDNN, ms^2^			0.02
Mean	1.68±0.21	1.87±0.42	
Range	0.85–1.95	0.97–2.10	
No.	8640	8640	
Log_10_ r-MSSD, ms^2^			0.02
Mean	1.21±0.24	1.54±0.33	
Range	0.52–1.72	0.64±1.87	
No.	8640	8640	
Coffee consumption, n			–
No	15	15	
Female	7	7	
Male	8	8	
Yes	15	15	
Female	8	8	
Male	7	7	

a Difference between daytime and nighttime participants was tested by t-test.

**Table 2:** Summary statistics for indoor air pollution levels, noise and meteorological variables (mean ± standard deviation).

	Daytime participants	Nighttime participants	P value^a^
Indoor air pollutants and noise			
PM_2.5_ 1-hour mean, µg/m^3^	26.5±9.5	22.1±4.3	<0.01
Interquartile Range	18.6	15.6	
Range	17.5–52.0	16.4–39.1	
No.	8640	8640	
Total VOCs 1-hour mean, ppb	47.5±22.4	41.1±15.6	<0.01
Interquartile Range	26.4	23.9	
Range	10–115.5	10–82.3	
No.	8640	8640	
CO_2_ 1-hour mean, ppm	125±59	125±60	0.46
Interquartile Range	80.6	79.8	
Range	25–450	21–421	
No.	8640	8640	
CO 1-hour mean, ppm	0.9±0.3	0.8±0.4	0.45
Interquartile Range	0.5	0.5	
Range	0.4–1.9	0.4–1.4	
No.	8640	8640	
Noise 1-hour mean, dBA	52.0 ± 10.1	42.5 ± 6.4	<0.01
Range	30.0–98	30.0–92	
No.	8640	8640	
Meteorological variables			
Temperature, °C	25.2±3.0	24.6±2.8	0.23
Range	19.8–30.5	18.9–29.3	
No.	8640	8640	
Relative humidity, %	56.4±8.2	55.7±7.8	0.34
Range	29.4–75.1	40.1–82.3	
No.	8640	8640	

a Difference between daytime and nighttime participants was tested by t-test.

Table 3 lists the associations between indoor air pollutants and HRV indices among daytime and nighttime participants. With sex, age, BMI, order of the visit, hour of day, temperature, relative humidity and noise being adjusted in our mixed-effects models, indoor PM_2.5_ and total VOCs exposures significantly decreased SDNN and r-MSSD for both the daytime participants and the nighttime participants. By contrast, CO exposures were not associated with SDNN or r-MSSD in our study subjects. The greatest decreases in SDNN and r-MSSD occurred with indoor PM_2.5_ and total VOCs exposures at 4-hr time-weighted moving averages (IQR = 14.2 µg/m^3^ and 22.8 ppb) in the nighttime participants. We examined the time course of indoor air pollutants exposures only up to 4-hr time-weighted moving averages because available data became substantially decreased for calculating time-weighted moving averages greater than 4 hr.

**Table 3:** Percentage changes (95% confidence interval)^a^ in heart rate variability for interquartile increase in indoor pollutants exposures estimated by mixed effects models.

		Daytime participants			Nighttime participants		
	Exposure matrix	PM_2.5_	Total VOCs	CO	PM_2.5_	Total VOCs	CO
SDNN	1-hour moving	−0.62*	−1.26	1.50	−1.65*	−1.39	−1.21
		(−1.01, −0.23)	(−3.46, 0.94)	(−3.55, 6.55)	(−2.70, −0.60)	(−3.39, 0.61)	(−3.00, 0.58)
	2-hour moving	−1.17*	−0.40	−0.02	−2.50*	−2.00	1.05
		(−2.13, −0.21)	(−1.13, 0.33)	(−1.23, 1.19)	(−3.55, −1.45)	(−4.19, 0.19)	(−2.04, 4.14)
	3-hour moving	−2.75*	−1.50*	−0.88	−3.75*	−1.88*	0.09
		(−3.00, −2.50)	(−2.11, −0.89)	(−5.69, 3.93)	(−4.22, −3.28)	(−3.17, −0.59)	(−1.52, 1.70)
	4-hour moving	−3.05*	−2.50*	−1.00	−4.78*	−2.86*	−1.70
		(−5.40, −0.70)	(−4.45, −0.55)	(−2.19, 0.19)	(−6.13, −3.43)	(−3.81, −1.91)	(−3.77, 0.37)
r-MSSD	1-hour moving	1.02	1.22	0.20	−1.33*	−0.12	−0.11
		(−3.11, 5.15)	(−4.50, 6.94)	(−2.47, 2.87)	(−2.00, −0.66)	(−2.12, 1.88)	(−1.08, 0.86)
	2-hour moving	−1.20*	−0.68	1.02	−1.98*	−1.35	−1.07
		(−2.15, −0.25)	(−9.03, 7.67)	(−2.23, 4.27)	(−2.92, −1.04)	(−3.25, 0.55)	(−4.44, 2.30)
	3-hour moving	−1.45*	−1.11*	1.80	−2.25*	−2.10*	−0.36
		(−2.01, −0.89)	(−2.75, −0.53)	(−4.89, 8.49)	(−4.12, −0.38)	(−3.10, −1.10)	(−2.65, 1.93)
	4-hour moving	−2.00*	−2.09*	−0.02	−3.23*	−3.17*	1.55
		(−3.00, −1.01)	(−3.17, −1.01)	(−2.00, 1.96)	(−4.88, −1.58)	(−4.65, −1.69)	(−0.55, 3.55)

a Coefficients are expressed as % changes for interquartile changes in indoor air pollutants exposures in models adjusting for sex, age, BMI, order of the visit, hour of day, temperature, relative humidity and noise. * p<0.01.

In mixed-effects models in which 4-hr time-weighted moving averages pollutants predicted HRV indices, we found the effect of indoor PM_2.5_ and total VOCs to be modified by coffee consumption and working time (Table 4). Participants with coffee consumption showed no significant change in HRV indices, whereas participants without coffee consumption showed significant changes in SDNN and r-MSSD associated with indoor air pollutants at 4-hr time-weighted moving averages (P value for interaction<0.05). We also found stronger effects of indoor PM_2.5_ on HRV indices among participants working at nighttime than at daytime (P value for interaction<0.05).

**Table 4:** The effect modification (95% confidence interval)^a^ of association of indoor air pollutants at 4-hr time-weighted moving averages with heart rate variability by coffee consumption and working time.

	SDNN		r-MSSD	
	PM_2.5_	Total VOCs	PM_2.5_	Total VOCs
Coffee consumption				
Yes	1.21 (−2.13, 4.55)	−0.01 (−1.89, 1.87)	−0.11 (−1.96, 1.74)	2.02 (−1.19, 5.23)
No	−3.21 (−4.02, −2.40)*	−4.25 (−5.09, −3.41)*	−2.99 (−3.20, −2.78)*	−3.15 (−4.44, −1.86)*
P value for interaction	0.041	0.025	0.021	<0.001
Working time				
Daytime	−1.19 (−2.12, −0.26)*	−2.12 (−3.25, −0.99)*	−0.89 (−1.62, −0.16)*	−1.56 (−2.25, −0.87)*
Nighttime	−3.54 (−4.31, −2.77)*	−2.96 (−3.56, −2.36)*	−2.87 (−4.02, −1.72)*	−2.06 (−3.16, −0.96)*
P value for interaction	0.036	0.26	0.045	0.19

a Coefficients are expressed as % changes for interquartile changes in indoor air pollutants exposures in models adjusting for sex, age, BMI, order of the visit, hour of day, temperature, relative humidity and noise.

* p<0.01.

We further investigated the associations between indoor air pollutants and HRV indices among daytime and nighttime participants with or without coffee consumption (Table 5). With sex, age, BMI, order of the visit, hour of day, temperature, relative humidity and noise being adjusted in our models, indoor PM_2.5_ and total VOCs exposures significantly decreased SDNN and r-MSSD for both the daytime and the nighttime participants without coffee consumption. By contrast, pollution effects were lowest for the daytime participants with coffee consumption.

**Table 5:** Percentage changes (95% confidence interval)^a^ in heart rate variability for interquartile increase in indoor pollutants at 4-hr time-weighted moving averages among participants with or without coffee consumption during the study.

	Participants with coffee consumption		Participants without coffee consumption	
	PM_2.5_	Total VOCs	PM_2.5_	Total VOCs
Daytime				
SDNN	−1.01*	−1.48	−3.15*	−2.28*
	(−1.80, −0.21)	(−5.41, 2.45)	(−5.01, −1.29)	(−4.00, −0.56)
r-MSSD	−0.89	−1.01	−2.97*	−2.06*
	(−2.01, 0.23)	(−3.76, 1.74)	(−3.88, −2.06)	(−3.65, −0.47)
Nighttime				
SDNN	−1.99*	0.89	−5.67*	−3.99*
	(−2.74, −1.24)	(−1.00, 2.78)	(−8.16, −3.18)	(−5.54, −2.44)
r-MSSD	−1.77*	−1.43*	−4.69*	−4.91*
	(−3.01, −0.53)	(−2.11, −0.75)	(−6.40, −2.98)	(−5.13, −4.69)

a Coefficients are expressed as % changes for interquartile changes in indoor air pollutants exposures in models adjusting for sex, age, BMI, order of the visit, hour of day, temperature, relative humidity and noise.

* p<0.01.

## Discussion

We found statistically significant association between indoor air pollutants exposures and decreased SDNN and r-MSSD among convenient store workers. When the working time was separated into the daytime (0700–1500 hours) and nighttime (2300-0700 hours) periods, the most pronounced decreases in HRV indices were seen at nighttime. Furthermore, we observed statistically significant effects of indoor air pollutants on HRV decreases among participants without coffee consumption compared with those among participants with coffee consumption. Few studies have investigated the association between air pollution exposures and HRV indices [16], [17] in nighttime, the majority have examined effects of ambient air pollution on autonomic function [7]–[9], [18] to date. Our study is the first to demonstrate decreases in HRV indices over daytime and nighttime subsequent to occupational exposure to indoor air pollution among convenient store workers with/without coffee consumption.

Although direct comparison between our study findings and previous studies is not feasible due to the differences of study subject and environmental exposure characterization, the direction of associations in our study is consistent with the inverse exposure-response association found in previous studies of particulate air pollution on HRV indices in nighttime. In a panel of 36 male boilermaker welders, elevated PM_2.5_ levels were associated with decreased 5-min SDNN and- 5-min r-MSSD in night periods (2400-0700 hours) [17]. A similar association was observed with 10-min SDNN in a panel of 10 nonsmoking, male highway patrol troopers while working a 3 p.m. to midnight shift [16]. Moreover, an inverse association between 6-hr SDNN in nighttime (2400-0600 hours) and same-day PM_10_ (particulate matter with an aerodynamic diameter ≤ 10 µm) exposure was observed among seven cardiac patients [19]. Such findings are consistent with the decreases in 5-min SDNN and 5-min r-MSSD that we observed in nighttime convenient store workers following occupational exposure to indoor PM_2.5_ exposure. It should be noted that sleep is an important modulator of HRV specific to the stages of sleep [20]. The observed associations between nighttime HRV indices and PM exposures in previous studies [16], [17], [19] covering the sleep period may be related to stage-related alterations in vagal tone. In the present study, all participants were awake and working in daytime and nighttime; thus, the observed decreases in HRV indices may directly result from pollution-related changes during the study.

An important finding of the present study is the observation that effects of indoor air pollutants on HRV indices were stronger when participants worked during nighttime compared with daytime periods of work, despite the fact that job assignments were similar during these two shifts. Shiftwork has direct effects on cardiac autonomic activity [21] and is associated with increased risk of coronary heart disease [22]. Some studies have reported that important cardiovascular risk factors, including hypertension, low- high density lipoprotein cholesterol and high triglyceride levels are higher for shift workers than for daytime workers [23], [24]. Among a panel of 22 blue-collar workers in a steel company, HRV indices were lower when workers worked at night compared with morning and evening periods of work [25]. Accordingly, nighttime work may potentially increase the vulnerability to the occurrence of cardiovascular diseases and susceptibility to air pollution. These findings may explain stronger effects of indoor air pollutants on HRV indices among participants working at nighttime than at daytime. However, we may not completely avoid healthy worker effect even though we used selection criteria to recruit similar participants for daytime and nighttime groups in this study. Such effect may have resulted in the lack of significant differences in the effects of indoor air pollution on HRV indices.

Another interesting finding in our study is that participants with coffee consumption had lower pollution-effects on decreased HRV indices compared to participants without coffee consumption during the study. Caffeine ingestion in a beverage has been demonstrated to enhance modulation of parasympathetic nerve activity in humans [26]. A recent study indicated that espresso coffee consumption influences parasympathetic activity and then increases HRV indices in 20 young, healthy sedentary subjects. Furthermore, this study showed that a cup of espresso coffee didn’t modify blood pressure, suggesting that coffee consumption didn’t induce hypertensive effects [15]. In the present study, we have observed that participants with coffee consumption had significantly higher values of SDNN (mean ± standard deviation of daytime and nighttime = 1.77±0.35 and 2.01±0.45 ms^2^) and r-MSSD (daytime = 1.32±0.27 ms^2^; nighttime = 1.62±0.40 ms^2^) than the participants without coffee consumption (daytime and nighttime SDNN = 1.59±0.25 and 1.74±0.29 ms^2^; daytime and nighttime r-MSSD = 1.11±0.18 and 1.46±0.30 ms^2^). Although direct evidence is needed to demonstrate that coffee consumption can modify indoor air pollution effects on autonomic function in young healthy subjects, the present study is the first experiment suggests some caution in the use of coffee for young healthy workers with pollution-induced decreases in HRV indices. Further intervention studies are needed to explore whether coffee consumption can lower effects of indoor air pollution on alteration of cardiac rhythm.

The activation of the autonomic nervous system by noise has been reported to play an important role in producing disturbances of physiological functions of the cardiovascular system [27]. In the present study, no significant association was observed between noise and any of HRV indices in our models. Such result was expected, as environmental status had no significant effects on noise levels in daytime (8-hr time-weighted average level = 51 dBA) and nighttime (46 dBA). Therefore, the observed declines in HRV may result from pollution-related changes during the study.

### Conclusions

The present study demonstrated decreases in 5-min SDNN and 5-min r-MSSD subsequent to indoor air pollutants exposures in a panel of 60 young healthy convenient store workers. Furthermore, we found that the decreases in HRV indices were most pronounced in nighttime workers without coffee consumption. More studies is needed to elucidate whether this is because nighttime incorporates the lag effect of ambient air pollution or coffee is a confounding factor and mask the effect of indoor air pollution on HRV indices in young healthy workers. Finally, our study further supports the cardiotoxicity of indoor particulate exposure. Future study is needed to investigate which of the particulate constituents is responsible for decreases in nighttime HRV.
